# Altered Social Processing as a Potential Mechanism of BPD Risk in Girls with ADHD: A Call for Multi-Method Developmental Research

**DOI:** 10.20900/jpbs.20250001

**Published:** 2025-01-06

**Authors:** Helena F. Alacha, Carla Sharp, Autumn Kujawa, Dara E. Babinski

**Affiliations:** 1 Department of Psychiatry and Behavioral Health, Penn State College of Medicine, Hershey, PA 17033, USA; 2 Department of Psychological and Brain Sciences, University of Louisville, Louisville, KY 40292, USA; 3 Department of Psychology, University of Houston, Houston, TX 77204, USA; 4 Department of Psychology and Human Development, Vanderbilt University, Nashville, TN 37203, USA

**Keywords:** ADHD, BPD, biosocial model, developmental mechanisms, social processes

## Abstract

Childhood attention-deficit/hyperactivity disorder (ADHD) symptoms prospectively predict the development of borderline personality disorder (BPD) symptoms in adolescence and adulthood; adult women with BPD, in particular, often retrospectively report childhood ADHD symptoms. However, little is known about specific developmental pathways and mechanisms that contribute to this sequential comorbidity. Herein we outline a call for multi-method developmental research examining altered social processing as a potential mechanism underlying risk for BPD in girls with ADHD. We review relevant developmental psychopathology theory, describe recent empirical work, and outline steps for future work with the goal of promoting continued research focused on reducing the personal and societal burden associated with ADHD and BPD.

## INTRODUCTION

Borderline personality disorder (BPD) is one of the most stigmatized mental health disorders, characterized by profound social dysfunction, risk for suicide, and long-term and costly mental health care [1]. Although traditionally diagnosed among adults [2], disproportionately among women, there is accumulating evidence that the developmental origins of BPD can be traced back to childhood [1,3]. In particular, girls with ADHD are at elevated risk for the development of BPD [4–6]. As many as 60% of women with BPD retrospectively report a childhood diagnosis of ADHD, and prospective longitudinal studies show that as many as 20% of girls with ADHD develop BPD by adulthood [4]. Even in childhood and adolescence, girls with ADHD demonstrate elevated levels of BPD features that are uniquely associated with poor outcomes [4,7,8]. Yet very little empirical research has examined early mechanisms and developmental pathways to risk for BPD in girls with ADHD, missing opportunities to mitigate risk for BPD across the lifespan. In this Viewpoint, we review developmental psychopathology theory, describe recent empirical work, and outline steps for future work in order to promote continued research focused on reducing the risk for BPD among girls with ADHD.

## DEVELOPMENTAL PATHWAYS LINKING ADHD AND BPD

**Figure 1.**
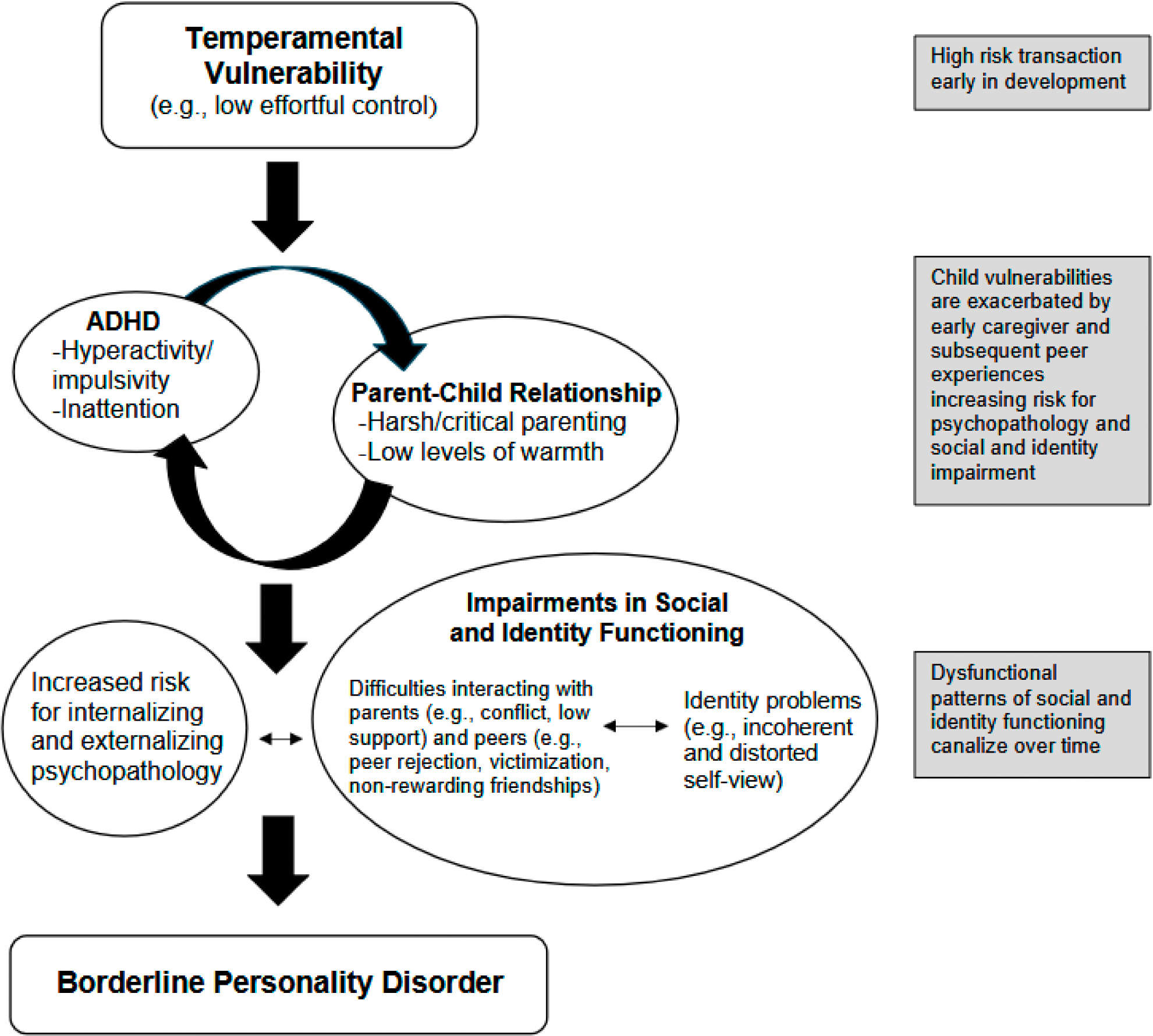
Proposed model of ADHD in childhood as a precursor to BPD in adulthood.

Prominent developmental models of BPD, including the biosocial model, provide a useful framework to examine early risk for BPD among girls with ADHD. These models converge in proposing that BPD emerges from dynamic transactions between a child’s biological and temperamental vulnerabilities, such as impulsivity, negative affectivity, and high emotional sensitivity, and invalidating environmental experiences [3,9]. The biosocial model has high relevance to girls with ADHD (Figure 1), many of whom face a range of invalidating environmental experiences, such as harsh and critical parenting, peer rejection and victimization, as well as abuse and other traumatic life events beginning early in development (e.g., [3,10]). According to the biosocial model, these experiences enhance risk for additional and more severe psychopathology, including oppositional defiant disorder, anxiety, and depression, which, in turn, contribute to worsening, and more entrenched social impairment [3]. Over time, dynamic transactions between psychopathology and negative environmental experiences cause further social impairment, impeding identity development and adolescent and adult relationships, ultimately increasing risk for the development of BPD [3,8].

There has been some research considering impulsivity and emotion dysregulation as mechanisms linking ADHD and BPD [7]. However, social processes are also highly relevant to understanding risk for BPD [2,11]. Some emerging evidence suggests that negative peer experiences, such as having low quality friendships and peer rejection and victimization, may enhance risk for BPD among girls with ADHD [4,5,12]. Within the biosocial model, negative social experiences are posited to exacerbate risk for BPD. Additionally, recent models of personality pathology and diagnosis, including the Alternative Model of Personality Disorders [13] and the International Classification of Disorders—11th Edition [14], identify social impairment, rather than impulsivity and emotion dysregulation, to be the core criterion of personality pathology. Marked cognitive, affective, and social changes occur in the transition to adolescence that are associated with heightened salience of social information [15], particularly peer-related information [16], which may contribute to increased risk for BPD. Thus, examining social processes during adolescence may be critical for identifying early opportunities to address the risk of BPD in girls with ADHD. While it is clear that social experiences in the environment impact risk for BPD [8,17], the internal processes relevant to understanding how girls with ADHD perceive and interpret this social information are not well understood.

## ALTERED PROCESSING OF SOCIAL REJECTION AND ACCEPTANCE CUES IN ADHD AND BPD

Prominent models of social processing posit that social information is processed in a set of stages, with earlier stages—including encoding and interpreting social cues—influencing later stages of social processing, such as selecting and enacting behavioral responses to social cues [18]. Sensitivity to social rejection has been a primary clinical focus of BPD and deficits in processing social rejection cues have been implicated in BPD and girls with ADHD [2,19]. However, similar social processing deficits are identified among individuals with depression and anxiety [20,21], and it is not clear whether sensitivity to rejection is a unique mechanism underlying risk for BPD. Instead, there is accumulating evidence pointing to distinct alterations in processing social rewards, such as acceptance cues, in risk for BPD. To date, self-report and behavioral methods have been used to identify deficits in the later stages of social processing. For example, adults with BPD report lower levels of social connection relative to those without BPD after being included [22,23] and are less likely compared to those without BPD to trust others and act cooperatively after receiving acceptance feedback [24]. Deficits in earlier stages of social processing are also likely, as adults with BPD report having lower expectations for social acceptance [23]. However, self-report methods are limited in discerning alterations in the early encoding and interpretation of social cues, as social processing is posited to be a mostly automatic process [18], and adults with BPD may have limited self-awareness of these processes [25].

## MULTI-METHOD ASSESSMENT OF SOCIAL PROCESSES UNDERLYING ADHD AND BPD

The National Institute of Mental Health (NIMH) RDoC Social Processes domain offers an innovative framework to examine how girls with ADHD perceive, interpret, and respond to social cues across the self-report, behavioral, and neural levels. Neural measures hold promise in advancing extant self-report and behavioral work to further elucidate deficits in social processing among youth with ADHD at risk for BPD. Innovative research using functional magnetic resonance imaging (fMRI) suggests that adults with BPD demonstrate enhanced reactivity to inclusion feedback in the dorsomedial prefrontal cortex [26,27], a region implicated in a number of cognitive functions, including emotional conflict monitoring and self-referential mentalizing of social knowledge. While this enhanced reactivity is speculated to reflect conflict between the desire to be socially accepted and the internal belief of those with BPD that others will reject them [27], fMRI methods lack temporal specificity, leaving questions about alterations in more immediate stages of social acceptance processing in BPD.

Event-related potentials (ERPs), derived from electroencephalograms (EEG), are well-established neural measures ideally suited to examine alterations in the earliest stages of social processing. ERPs are known for their excellent temporal resolution, reliability, and economical assessment across development [28–30] and have been examined as indicators of individual differences in social processes, such as responses to social rejection and acceptance [31]. The reward positivity (RewP) is an ERP that may be particularly relevant for examining processing of social acceptance cues and the risk of BPD among girls with ADHD. The RewP is a frontocentral ERP component that appears approximately 300 ms after stimuli onset that reflects activation of reward-related brain regions, including the ventral striatum and medial prefrontal cortex [29]. The RewP has been associated with self-report and behavioral measures of reward responsiveness [32,33], potentially reflecting individual differences in reward sensitivity and approach motivation. RewP has been implicated as a neural marker of both monetary and social reward responsiveness [31,32,34].

## RECENT FINDINGS AND A ROADMAP FOR FUTURE RESEARCH

Our recent work highlights potential value in examining neurophysiological processing of social cues, and specifically the RewP, in the development of personality dysfunction. In order to study early risk for personality disorders, we measured personality pathology dimensionally and recruited a sample of 109 girls (*M*_age_ = 12.21 years, *SD* = 1.21) [35]. Most girls were identified based on a history of mental health problems, including ADHD (approximately 12%) as well as anxiety, depression, and conduct problems; autism spectrum disorder (ASD), bipolar disorder, schizophrenia or other psychotic disorders were exclusionary. The sample also included 30 girls without mental health problems. Girls completed an innovative peer interaction task that has been shown to reliably elicit an enhanced RewP to peer acceptance cues relative to rejection cues [31,32]. A regression analysis covarying symptoms of depression, anxiety, ADHD, oppositional defiant disorder, and conduct disorder revealed a modest but significant association between enhanced RewP to social acceptance (while accounting for response to social rejection) and higher levels of personality pathology [35]. While this finding does not specifically address the risk of BPD among girls with ADHD, it suggests that future work examining neurophysiological processing of social cues in this population is worthwhile and may reveal meaningful insights into the processes underlying risk for BPD. This study, combined with additional studies that our team is undertaking, will incrementally advance our understanding of the social processing mechanisms underlying risk for BPD among girls with ADHD. We will examine differences in neurophysiological processing of peer feedback using the innovative peer interaction task [31,32] described above in early adolescent girls with and without ADHD (R21MH124027), and in early adolescent girls and boys with and without mental health problems (R21MH125052; R01MH132620). It is anticipated that findings will identify potentially modifiable intervention targets to inform the development of interventions to address risk for BPD among girls with ADHD before the emergence of more high-risk and intractable behaviors.

## Data Availability

Data sharing is not applicable to this article as no datasets were generated or analyzed during the current study. Data for the described projects is ongoing. The datasets generated from this work will be made available in the National Institute of Mental Health Data Archive (NDA).
